# A comparative review on the anti-nutritional factors of herbal tea concoctions and their reduction strategies

**DOI:** 10.3389/fnut.2022.988964

**Published:** 2022-10-05

**Authors:** Neeta Pathaw, Konjengbam Sarda Devi, Redina Sapam, Jyotsana Sanasam, Sapam Monteshori, Sumitra Phurailatpam, Huirem Chandrajini Devi, Wangkhem Tampakleima Chanu, Baby Wangkhem, Naorem Loya Mangang

**Affiliations:** ^1^Indian Council of Agricultural Research, Research Complex for North Eastern Hill Region, Imphal, Manipur, India; ^2^Multi Technology Testing Centre and Vocational Training Centre, College of Agriculture, Central Agricultural University, Imphal, Manipur, India; ^3^College of Agriculture, Central Agricultural University, Imphal, Manipur, India; ^4^Department of Agriculture, Himalayan University, Itanagar, Arunachal Pradesh, India

**Keywords:** anti-nutritional, herbal tea, underutilized plants, saponins, phytic acid, alkaloids, anti-nutrients

## Abstract

Tea is an important beverage consumed worldwide. Of the different types of tea available, herbal tea is an important beverage consumed owing to its popularity as a drink and stress relieving factors, several different herbal concoctions made from seeds, leaves, or roots are currently consumed and sold as herbal teas. The herbal teas are not the usual tea but “tisanes.” They are caffeine free and popular for their medicinal property or immune boosters. Herbal tea formulations are popularly sold and consumed by millions owing to their health benefits as they are rich in antioxidants and minerals. However, plants are also known to contain toxic and anti-nutritional factors. Anti-nutritional factors are known to interfere with the metabolic process and hamper the absorption of important nutrients in the body. These anti-nutritional factors include saponins, tannins, alkaloids, oxalates, lectins, goitrogens, cyanogens, and lethogens. These chemicals are known to have deleterious effects on human health. Therefore, it is important to understand and assess the merits and demerits before consumption. Also, several techniques are currently used to process and reduce the anti-nutrients in foods. This review is focused on comparing the contents of various anti-nutritional factors in some underutilized plants of North-East India used as herbal tea along with processing methods that can be used to reduce the level of these anti-nutrients.

## Introduction

Plants and plant products have been a major source of dietary consumption for humans. Apart from being consumed for nutritional sources, they have also been used for medicinal purposes. The writings of many ancient civilizations suggest the extensive use of plants or plant products as herbal concoctions to treat various ailments. These herbal drinks or beverages later gained popularity owing to their health benefits. Tea is one of the most popular beverages consumed by millions worldwide. It is known to have many health benefits viz., anti-oxidative, anti-hypertensive, and hypolipidemic activities (1). Tea made from leaves of the plant Camellia sinensis dates centuries back and consists of black and green drinks with health benefits contributed by polyphenols such as catechins and theaflavin (2). Along with all the health benefits tea also contains caffeine, hence as a healthy alternative low calorie and decaffeinated traditional herbal drinks are gaining popularity (3). Herbal teas are made with boiling or water infusions of leaves, flowers, roots, barks, etc. (4). Herbal teas are actually mixtures of several ingredients, and are more accurately known as “tisanes” which is derived from the Greek word “ptisane” that means crushed barley. Tisanes are made from combinations of dried leaves, seeds, grasses, nuts, barks, fruits, flowers, or other botanical elements that give them their taste and provide the benefits of herbal teas (5). Herbal Tea comes under the list of beverages with aroma, taste, and healing properties. It has a variety of health benefits, including relaxation, decreasing body temperature, alleviating a poor stomach, and reducing fluid retention in the body (6). It is made by steeping various herbs in water and serving it hot or cold, depending on the user's desire. Herbal teas are popular for their therapeutic and invigorating characteristics that aid in body cleansing and strengthening the immune system. Some of the most popular herbal teas are chamomile, ginger, cardamom, and peppermint with each one having therapeutic potential (4). The past few decades have seen an increased use of plants with medicinal properties being consumed as herbal drinks worldwide. Plants or plant products form an essential part of people's lives as a source of fulfilling their nutritional needs. In spite of plants providing nutrition, it is also a source of many anti-nutrients including tannins, steroids, alkaloids, saponins, phytic acid, flavonoids, and cyanogens. These anti-nutrients interfere with the assimilation of nutrients or inhibit the utilization of nutrients such as proteins and minerals such as iron and zinc (7). The anti- nutritional factors that are known to lower the nutritional value of food can also be reduced by processing or following preparations such as heating, steaming, fermenting, cooking, and boiling. These processing techniques followed traditionally not only aid in storing the plant products but also help in reducing the anti-nutrients. The current paper is an attempt to highlight the use of some of the most popular herbal drinks prepared from some underutilized as well as commonly consumed plants from the north-eastern part of India with the emphasis on the anti-nutrients contents and strategies to reduce the effect of these anti-nutrients.

## Underutilized plants as beverages and their processing

**Figure 1:**
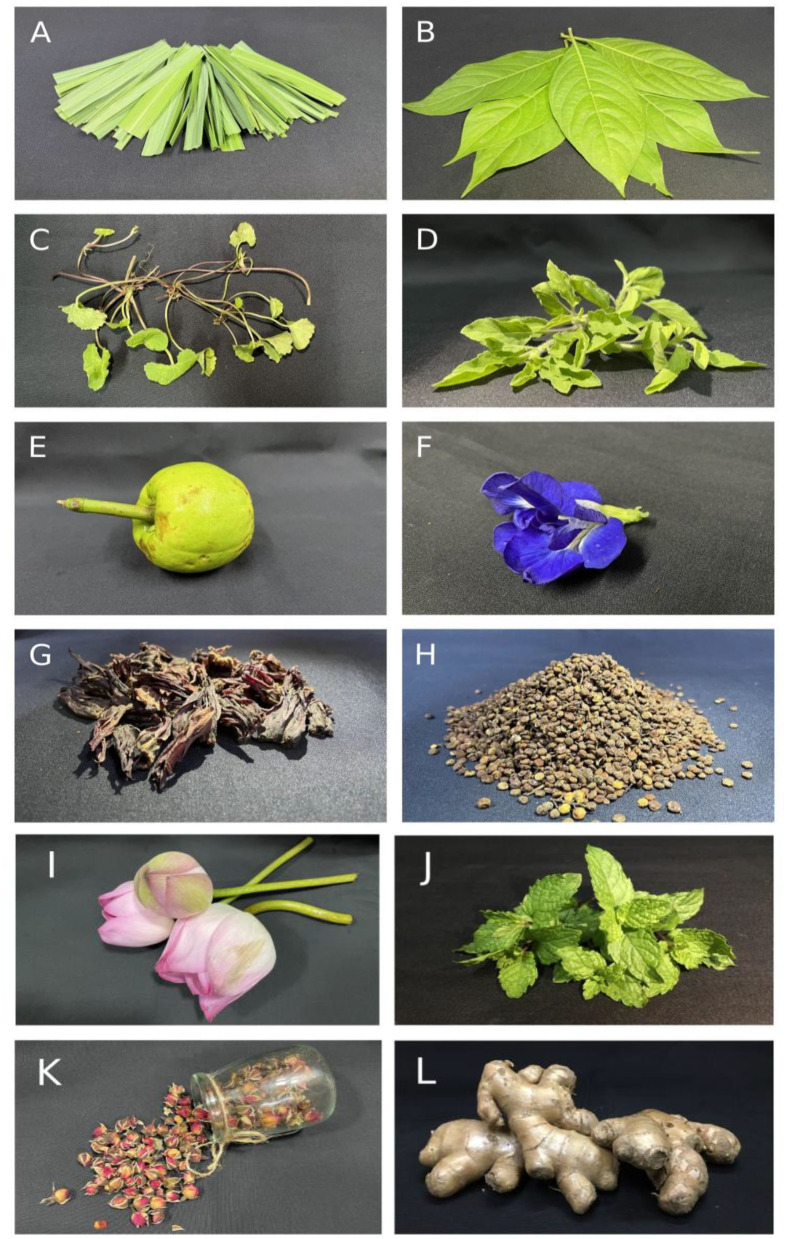
Pictures of different plants that are consumed as herbal tea studied in the current review. **(A)** Lemongrass (*Cymbopogon citratus*); **(B)** Nongmangkha (*Phlogacanthus thyrsiformis*); **(C)** Peruk (*Centella asiatica*); **(D)** Holy Basil/Tulsi (*Ocimum sanctum*); **(E)** Heibung (*Garcinia pedunculata*); **(F)** Blue pea (*Clitoria ternatea*); **(G)** Roselle (*Hibiscus sabdariffa*); **(H)** Heimang (*Rhus chinensis*). **(I)** *Nelumbo nucifera* (Lotus), **(J)** *Mentha spicata* (Spearmint), **(K)** *Rosa damascena* (Rose), and **(L)** *Zingiber officinale* (Ginger).

Herbal teas are the primary sources of dietary antioxidants in many cultures, of which polyphenolic compounds, in addition to vitamins and carotenoids, have been the focus of the scientific community for the past few decades (8). The World Health Organization in its 2014–2023 strategy has prioritized the screening of plants with medicinal potential with the aim to capitalize on the use of traditional medicine as a source for providing an effective and low cost alternative healthcare coherent to their cultural practices. As these strategies become policies that will affect healthcare practices in the future, therefore, it has become even more pertinent to address issues related to the safe and effective use of these medicinal plants as herbal drinks. As one of the biodiversity hotspots, the North Eastern region of India houses a plethora of medicinal plant species, many of which are commonly consumed. The region, having vast forest cover, is also home to many underutilized plants as a nutritional source or consumed as beverages. Figure 1 shows some of the commonly consumed beverages in North east India. viz., *Cymbopogon citratus* (Lemongrass), *Phlogacanthus thyrsiformis* (Nongmangkha), *Centella asiatica* (Peruk), *Oscimum sanctum* (Basil), *Garcinia pedunculata* (Heibung), *Clitoria ternatea* (Blue pea), *Hibiscus sabdariffa* (Roselle), *Rhus chinensis* (Heimang), *Nelumbo nucifera* (Lotus), *Mentha spicata* (Spearmint), *Rosa damascena* (Rose), and *Zingiber officinale* (Ginger).

*Cymbopogon citratus* is a tropical herb of the Poaceae family. The identified compounds in *C. citratus* are mainly terpenes, alcohols, ketones, aldehydes, and esters. Some of the reported phytoconstituents are essential oils that contain citral α, citral β, nerol geraniol, citronellal, terpinolene, geranyl acetate, myrcene, and terpinyl methyl heptenone. The plant also contains phytoconstituents such as flavonoids and phenolic compounds, which consist of luteolin, isoorientin 2′-O-rhamnoside, quercetin, kaempferol, and apigenin. Studies indicate that *C. citratus* possesses various pharmacological activities such as anti-amoebic, antibacterial, anti-diarrheal, anti- filarial, anti-fungal, and anti-inflammatory properties. Various other effects such as antimalarial, anti- mutagenicity, anti-mycobacterial, antioxidants, hypoglycemic, and neurobehavioral have also been studied (14).

*Phlogacanthus thyrsiformis* (Nongmangkha) is a shrub belonging to the Acanthaceae family. It is an evergreen shrub that grows to a height of 2.4 m, leaves are normally 13–35 cm long, oblanceolate, elliptic-oblong, acute, or acuminate. The flowers are terminal elongated, thysoid panicles, up to 30 cm long, orange or brick red in color (32). The whole plant is extensively used for its great medicinal value like antipyretic, anti-diabetic, cough, colds, and anti-asthma. The inflorescence is consumed as vegetables and leaves are medicinal (8). The herbal tea is prepared from the dried leaf of *P. thyrsiformis*.

*Centella asiatica* is the herbaceous plant of the Apiaceae family. A major component of its bioactive constituents is asiaticoside, asiatic acid, madecassoside, and madecassic acid. The whole plant of *Centella* consumed as herbal tea has the properties of antioxidant activities, and treats gastrointestinal disease, gastric ulcer, asthma, wound healing, and eczema (41). Leaves of *C. asiatica* (L) are a rich source of valuable primary and secondary metabolites such as carbohydrates, tannins, steroids, terpenoids, alkaloids, flavonoids, cardiac glycosides, and saponins. *C. asiatica* accumulates large quantities of pentacyclic triterpenoid saponins, collectively known as centelloids. *Centella* is also rich in Vitamin C, Vitamin B1, Vitamin B2, niacin, carotene, and Vitamin A and is reported to possess various pharmacological activities such as antimicrobial activity, anticancer activity, wound healing activity, neuroprotective activity, immunomodulatory activity, anti-inflammatory activity, hepatoprotective activity, insecticidal activity, and antioxidant activity. *C. asiatica* plant possesses potential thrombolytic and antioxidant effects (9).

*Ocimum sanctum* (Basil) is an aromatic shrub in the basil family Lamiaceae also known as tulsi or holy Basil. It has been found to protect organs and tissues against chemical stress from industrial pollutants and heavy metals, and physical stress from prolonged physical exertion, ischemia, physical restraint, and exposure to cold and excessive noise (30). Tulsi has also been shown to counter metabolic stress through normalization of blood glucose, blood pressure, and lipid levels, and psychological stress through positive effects on memory and cognitive function and its anxiolytic and antidepressant properties (30).

*Garcinia pedunculata* (Heibung) belongs to the Clusiaceae family. Traditionally, it has been used for the treatment of asthma, bronchitis, coughs, dysentery, fever, and maldigestion (35). A variety of beneficial effects have been reported from the fruit extract, including antimicrobial, antioxidant, anti-inflammatory, hepatoprotective, nephroprotective, and cardioprotective properties (35). According to a recent study, the fruit of *G. pedunculata* contains phenolic compounds, flavonoids that produce total anti-oxidant activity, as well as anthocyanins and ascorbic acid. There are also several benzophenones, including pedunculo, garcinol, and cambogin, as well as organic acids, such as citric acid, hydroxycitric acid, hydroxycitric acid lactone, and oxalic acid (35).

*Clitoria ternatea* (Blue pea) is a plant from the Fabaceae family. The flowers are ornamental and are traditionally used as a food colorant. *C. ternatea* is also referred to as the butterfly pea, blue pea, aprajita, Cordofan pea, or Asian pigeonwings, and is commonly found throughout South East Asia. The bright blue petals from the flowers of the butterfly-pea plant have been used as an ingredient in herbal tea, caffeine-free, or tisane. *C. ternatea* flower extracts were found to possess antimicrobial, antioxidant, anti-inflammatory, cytotoxic, and antidiabetic activities, and also has been used as a memory enhancer, nootropic, anti-stress, anxiolytic, antidepressant, anticonvulsant, tranquilizer, and sedative agent (12). Various secondary metabolites including triterpenoids, flavonol glycosides, anthocyanins, and steroids have been isolated from the plant. Its extracts possess a wide range of pharmacological activities including antimicrobial, antipyretic, anti-inflammatory, analgesic, diuretic, local anesthetic, anti-diabetic, insecticidal, blood platelet aggregation-inhibiting, and for use as a vascular smooth muscle relaxing properties (42) (Table 1).

**Table 1:** List of herbal teas studied along with their important phytochemical constituents and health benefits as well as its major consuming countries.

**Scientific name (local name) & family**	**Health benefits**	**Plant part(s) used**	**Phyto chemicals**	**Concentration of Phytochemicals (mg/100 g)**	**Major consuming countries**	**References**
*Centella asiatica* (Peruk) & Apiaceae	Potential thrombolytic and antioxidant effects, antimicrobial, anticancer, wound healing, neuroprotective, immunomodulatory, anti-inflammatory, hepatoprotective, insecticidal, and antioxidant	Leaves and the whole plant	Tannins Alkaloids Flavonoids Phenolics Phytates Steroids Soluble oxalates Oxalates	1.11 ± 0.02 0.29 ± 0.18 120.73 ± 0.17 109.32 ± 0.23 16.851 188.974 N/A N/A	Southeast Asia, South Africa, and Madagascar	(9–11)
*Clitoria ternatea* (Blue pea) & Fabaceae	Antimicrobial, antioxidant, anti-inflammatory, cytotoxic and antidiabetic activities, and also has been used as a memory enhancer, nootropic, anti-stress, anxiolytic, antidepressant, anticonvulsant, tranquilizer, and sedative agent	Flower	Flavonoids Phenolics Steroids	N/A N/A N/A	South and Central America, West Indies, Madagascar, India, China, Philippines, and other tropical Asian countries	(12, 13)
*Cymbopogon citratus* (Lemongrass) & Poaceae	Anti-amoebic, anti-diarrheal, anti-filarial, anti- mycobacterial, antioxidants, hypoglycaemic, anti-inflammatory properties	Leaves	Alkaloids Phenolics Saponins Tannins Flavonoids	1.38 ± 0.02 3.58 ± 0.02 1.25 ± 0.14 15.60 ± 0.03 4.76 ± 0.02	Asia, South America, and Africa	(13–16)
*Garcinia pedunculata* (Heibung) & Clusiaceae	Antimicrobial, antioxidant, anti-inflammatory, hepatoprotective, nephroprotective, and cardioprotective properties	Fruits	Flavonoids Phenolics Saponins Tannins Alkaloids	23.38 80.14 N/A N/A N/A	Southeast Asia	(17)
*Hibiscus sabdariffa* (Sougri) & Malvaceae	Diuretic, choleretic, febrifugal and hypotensive, decreasing the viscosity of the blood, and stimulating intestinal peristalsis	Flower (calyces)	Phenolics Alkaloids Flavonoids Saponins Tannins	37.42 N/A N/A N/A N/A	India, Central America, West Indies, and Africa	(18, 19)
*Mentha spicata* (Spearmint) & Lamiaceae	Sedative, carminative, anti-spasmodic, hypoglycemic action, diuretic action, antimicrobial activity, antioxidant activity, breath problems, and anti-inflammatory action	Leaves	Phenolics Flavonoids Alkaloids Saponins	2.629 ± 0.15 N/A N/A N/A	Europe, western, and central Asia	(20, 21)
*Nelumbo nucifera* (Lotus) & Nymphaeaceae	Antioxidant, anti-inflammatory, anti-diabetic, anti-obesity and anti-cancer activities, immunosuppression in diabetic patients	Flower	Phenolics Alkaloids Flavonoids Tannins	351.08 ± 4.62 N/A N/A N/A	Southeast Asia and Middle east	(22–29)
*Ocimum sanctum* (Tulsi) & Lamiaceae	Anxiolytic and antidepressant properties	Leaves	Phenolics Flavonoids Terpenoids Steroids	16.5 4.49 N/A N/A	Southeast Asia	(30, 31)
*Phlogacanthus thyrsiformis* (Nomangkha) & Acanthaceae	Antipyretic, anti-diabetic, cough, colds, and anti-asthma	Leaves	Flavonoids Tannins Saponins Steroids	0.13 0.29 N/A N/A	North East Region of India	(8, 32–34)
*Rhus chinensis* (Heimang) & Anacardiaceae	Antiviral, antibacterial, anticancer, hepatoprotective, anti-diarrheal, and antioxidant activities	Fruits	Phenolics Flavonoids Tannins Steroids Oxalates	725 ± 2.64 57.33 ± 1.13 N/A N/A N/A	North East Region of India	(35, 36)
*Rosa damascena* (Rose) & Rosaceae	Antioxidants, anti-constipation, anti-depression, reduces gastrointestinal disorders, inflammation, respiratory problems, and menstrual bleeding	Flower and flower buds	Phenolics Flavonoids	165.16 81.35	Bulgaria, China, India, Iran, Morocco, Ukraine, Libya, south of France, south of Italy, south of Russia, and Turkey	(37)
*Zingiber officinale* (Ginger) & Zingiberaceae	Anti-inflammatory activity, cardiovascular effects	Rhizome	Saponins Phytates Oxalates Tannins	4.01 ± 0.07 0.28 ± 0.01 0.26 ± 0.002 0.02 ± 0.00	Asia, Africa, and West Indies	(38–40)

*Hibiscus sabdariffa* (Roselle) is a sub herb of the Malvaceae family, infusions of the leaves or calyces are regarded as diuretic, choleretic, febrifugal, and hypotensive, decreasing the viscosity of the blood, and stimulating intestinal peristalsis (18). The dried calyces are consumed as an herbal tea. It has a high content of bioactive compounds such as phenolic acids, flavonoids, polysaccharides, and anthocyanins. The calyx extract of *H. sabdariffa* was mainly composed of anthocyanins, which contributed to its antioxidant capacity. It has been reported that *H. sabdariffa* extract is able to exhibit anticancer activity through its ability to protect against oxidative damage in rat primary hepatocytes (43).

*Rhus chinensis* (Heimang) belongs to the Anacardiacae family. Commonly known as the Nutgall tree or Chinese sumac, it is a deciduous tree abundantly grown in China, Japan, and north eastern India. In Manipur, North-East India, it is popularly known as “Heimang.” The whole fruit and seed are traditionally used for their digestive properties and possess strong antiviral, antibacterial, anticancer, hepatoprotective, anti-diarrheal, and antioxidant activities (12, 32). It was found that the fruit of *R. chinensis* contained a high level of crude fiber, crude fat, and total titratable acidity. Ascorbic acid, malic acid, and citric acid were detected as the major organic acids (36).

*Nelumbo nucifera* (Sacred lotus) is an aquatic species belonging to Nelumbonaceae having herbal remedial benefits in every part of the plant (44). Widely used in Ayurveda and traditional medicines (22, 23, 45), the lotus is native to tropical and subtropical zones of Asia and almost all parts of it, such as flower, seed, leaf, stem, and root are edible (44) whose extract contains various phytochemicals, including alkaloids, flavonoids, phenolic acids, and steroids (22, 23, 45–49), which promote antioxidant (23, 45, 50) anti-inflammatory (51, 52), anti-diabetic (24), anti-obesity (25), and anti-cancer (26) activities. These biological activities are beneficial for individuals with declined immune functions, especially to the aged and advanced age people (53, 54), and immunosuppression in diabetic patients (27).

*Mentha spicata* (Spearmint) is an aromatic herb belonging to the family Lamiaceae. The leaves or powder of this aromatic herb can be used as a seasoning and flavoring herb or traditionally as an herbal tea (20). Various pharmacological properties have been found in the leaves, such as sedative, carminative, anti-spasmodic, hypoglycemic action, diuretic action, antimicrobial activity, antioxidant activity, breath problems, and anti-inflammatory action. Typically, *M. spicata* leaves are prepared as tea infusions to treat high blood pressure, menstrual irregularities, digestive issues, and respiratory problems (21, 54, 55). Spearmint essential oil has been used to treat digestive problems, obesity, asthma, coughing, and colds in traditional Arabic Palestinian medicine. Volatile oil, phenols, flavonoids, and lignans are abundant in *M. spicata* leaves (55).

*Rosa damascena* (Rose) belongs to the Rosaceae family and is one of the most important rose species widely used in the pharmaceutical and food industries and producing high-value essential oil. *R. damascena* is called Damask Rose in English and Gole Mohammadi in Persian (37, 56). Traditionally, *R. damascena* has been used to treat chest pain, constipation, depression, gastrointestinal disorders, inflammation, respiratory problems, and menstrual bleeding. Furthermore, *R. damascena* is one of the most potent sources of antioxidants, including phenolics, flavonoids, carotenoids, and anthocyanins (37).

*Zingiber officinale* (Ginger) belongs to the family Zingiberaceae and is also a major crop, grown primarily in India, China, and Nigeria. It is used worldwide as a spice, condiment, and herbal intact. The plant is upright ranging from 60 to 90 cm in height with a pseudo-stem and possesses perennial tuberous or rhizomatous roots (38). The characteristic odor and flavor of ginger are caused by a mixture of zingerone, shogaols, and gingerols, volatile oils that are composed of 1–3% of the fresh ginger weight and the constituents of the ginger plant have physiologic effects and also contain various phytochemicals having biological activities such as antimicrobial, antioxidant, and other pharmacological effects (38). The plant is used in traditional medicine for the treatment of several ailments such as Rheumatism, diabetes, snake bite, wounds, baldness, stomach disorder, toothache, bleeding, arthritis, respiratory disorders, and rash, in different parts of the world (39).

Various parts of the plant are used for the preparation of decoction and consumption as herbal tea. Leaves of lemongrass, nongmangkha, peruk, mentha, and basil, fruits of heibung, and heimang, flower, calyx, and flower buds of a blue pea, roselle, lotus, rose, respectively, the rhizome of ginger are used for herbal tea preparation; most of them belongs to under-utilized plants which are preferably consumed for its medicinal and soothing properties. The procedure of herbal tea processing includes drying, an essential thermal process. Oven (cabinet) drying still remains an attractive option for bulk drying of fragrant leaves such as coriander, olive mint, basil, thyme, bay leaves, and olive leaves (57–60). The drying process increases the shelf life by slowing microbial growth and, thus, preventing certain biochemical reactions that might alter the organoleptic characteristics. Drying can be carried out simply by air drying or using the aid of machines. Since air drying in humid weather can lead to spoilage and quality reduction, oven drying is preferred over it. It can take up to a week or more to dry herbs depending on the humidity in the air and the moisture content of the plant parts used for herbal tea. Previous studies have demonstrated that drying, an essential thermal process in producing herbal teas, may cause significant losses of bioactive ingredients and thereby decrease the health benefits and quality of the products (61, 62). Drying is a key step in the preservation of lemongrass leaves and processing of the leaves further into value-added products and oil extraction. The processing of lemon grass tea from freshly chopped green lemongrass using the sun, solar dryer, microwave (model P70B17L-T8) at 50 W, and oven (Prolab Instrument—model OTE 80) at 40, 50, 60°C (63, 64) showed the effect of drying on quality and sensory attributes of lemon grass (*Cymbopogon citratus*) tea whose results showed that the sensory properties, moisture content, ash content, pH and color of lemongrass samples differed with respect to the drying methods used. Reportedly, lemongrass tea from samples dried by oven at 40°C was the most preferred in color, aroma, taste, and overall acceptability and most suitable as the retention of appreciable sensory attributes is highest. This herb with its medicinal, therapeutic, and flavoring/culinary uses is very popular in Asian, Thai, and Vietnamese cooking. It can be used in fresh, dried, or powdered forms as seasonings and teas. Spearmint can be air dried (21) under shade for processing as herbal tea. The processing of roselle utilizes fruit and calyxes (dried) to be used as herbal tea. Although there have been some studies on the extraction of roselle using different types of solvent (65–67) and investigations into the drying processes for these materials (68, 69), comprehensive studies to establish a convenient procedure for drying and brewing to achieve high-quality roselle tea infusion are limited. The use of a common hot-air dryer for drying roselle and the classical brewing method was investigated by Nguyen and Chuyen (43), to find out the most suitable drying and brewing conditions in order to produce roselle tea with a high content of bioactive, antioxidant activity and sensory quality. The combination of drying at 80°C and steeping dried roselle for 30 min in 90°C hot water with a 1:10 solid-liquid ratio (g/ml) produces roselle tea with the highest content of beneficial ingredients. The processing of roses utilizes petals and flowerbuds/rosebuds (dried) to be used as herbal tea, and similarly, in Lotus, lotus petals are dried and used as herbal tea. Lotus petals are dried with all their phytochemicals intact at 60°C for 48 h (44). In Manipur, Elizabeth Yambem founded Manipur Dweller tea, which developed Heimang tea, Heibung tea, Peruk tea, and Nongmangkha tea (36). Since heimang is traditional medicine, which offers health benefits, formulating drinks/tea using heimang seeds and other value-added ingredients such as cinnamon, ginger, tea leaves, and sugar and salt for taste will improve the quality of the product eventually providing additional health benefits. Devi Heirangkhongjam and Singh Ngaseppam (36) reported that dried fruits of heimang are normally used as ingredients in local culinary preparations instead of fresh fruits which are usually dried and stored to avoid spoilage. The whole fruits were dried at 55 ± 5°C in a hot air oven to process them for future use. Heibung being a rich source of secondary metabolites including xanthones, flavonoids, benzophenones, lactones, and phenolic acids with wide range of biological and pharmacological activities (70) are consumed; it has been processed using the fruit, sliced into small pieces and seeds separated. The sliced pulp dried up, which could either be powdered or unpowdered, and then packed for a healthy cup of herbal tea. A herbal tea is made from whole plants of peruk, leaves of nongmangkha, and tulsi, which are dried and powdered before being packaged. Ginger rhizome are sliced and dried which are powdered as its processing method for consumption as herbal tea. The rhizome of *Zingiber officinale* dried in an oven at a steady temperature of 60°C and milled with an electric blender before being ground into powder are used for evaluation of phytochemicals (39, 40). Blue tea is prepared from blue pea flowers, flowers are harvested and oven dried at 45–50°C (13) and can be stored for use as herbal tea. The dried flowers can be ground and packed for decoction of herbal tea.

## Anti-nutritional factors

Anti-nutritional factors (ANFs) are substances that reduce digestion, utilization of nutrients, interfere with the absorption of biomolecules and hamper their bioavailability to human beings and monogastric animals, and may produce other adverse effects. The major anti-nutrients found in plant-based foods are phytates, tannins, alkaloids, lectins, oxalates, etc. (7). Phytate, being the most important among all, reduces the bioavailability of micronutrients such as iron and zinc. The anti-nutritional factors may be classified on the basis of their effects on the nutritional value and the biological response. Antinutritional factors may be broadly grouped into, (i) Factors with a depressive effect on protein digestion and on the utilization of protein, such as protease inhibitors, tannins, and saponins, (ii) Factors that affect mineral utilization, which includes phytates, (iii) Factors that stimulate the immune system and may cause a damaging hypersensitivity reaction, such as antigenic proteins, (iv) Factors with a negative effect on the digestion of carbohydrates, such as amylase inhibitors, phenolic compounds, and flatulence factors (7). Also, it can be presented as (a) Non-protein Amino Acids (Mimosine) as in Leucaena, (b) Glycosides (Saponins) as in Acacia, (c) Polyphenolic compounds (Tannins, Lignins) as in all vascular plants, (d) Alkaloids and Oxalate as in Acacia. Table 2 summarizes some anti-nutrients and their effects on us.

**Table 2:** List of anti-nutrients found in herbal tea and its effects.

**Anti-nutrient**	**Its effects**	***In vivo*** **/*in vitro***	**References**
Oxalate	• It can form insoluble salts which accumulates kidney stones • It can bind to calcium and prevent it from being absorbed	*In vivo*	(71)
Tannins	• It affects protein digestibility and leads to reduction of essential amino acids (by forming reversible and irreversible tannin-protein complexes between the hydroxyl group of tannins and the carbonyl group of proteins)	*In vitro*	(71)
Steroids	• Increase risk of cardiovascular events including stroke or heart attack	*In vivo*	(72)
Phytates	• It impedes the absorption of minerals like iron, zinc, magnesium & calcium and also inhibits enzymes like pepsin, trypsin etc. • It affects the gastrointestinal absorption of minerals which in turn lowers the bioavailability of the minerals	*In vivo*	(73, 74)
Saponins	• It affects the absorption of vitamin A and E as well as lipids • It can lead to hypoglycemia	*In vivo*	(75)
Alkaloids	• Alkaloids are mostly involved in neurotoxicity or cell signaling disruption	*In vitro*	(71)
Phenolics	• Decrease bioavailability of amino acids • Loss of appetite, breathing problems, and cardiac complications	*In vitro*	(76)
Flavonoids	• It chelates metals such as iron & zinc and reduces the absorption of these nutrients • They also inhibit digestive enzymes and may also precipitate proteins	*In vivo* and *in vitro*	(73)
Terpenoids	• It alters the carbohydrate metabolism and also generates toxic effects in liver and kidney	*In vivo*	(74)
Soluble oxalate	• It exerts its effects by binding calcium (Ca), magnesium (Mg), and other trace minerals such as iron (Fe), making them unavailable for assimilation	*In vivo*	(77)

### Tannins

Tannins are a group of polyphenols of ~500 Da molecular weight present in plants. Tannins are secondary metabolites found in abundance in leaves, fruits, and bark (78). They are known to form complexes with protein between the hydroxyl and carbonyl group of tannins and proteins, respectively, thereby affecting protein digestibility and leading to inhibition of the utilization of essential amino acids and minerals (79, 80). Green tea and grapes are known to be rich in tannins. These are also found in plenty in berry fruits, beverages, pomegranate, cocoa, and also some cereals and legumes (81, 82). Naturally, there are two types of tannin groups-(a) hydrolysable-composed of gallotannins and ellagitannins and (b) condensed consisting of proanthocyanidin. The legume-bran is rich in tannins and hence affects the digestibility of proteins by forming tannin-protein complexes (83). Previous reports have shown that tea by-products produced in the beverage industry contain high levels of crude protein (CP, 220–350 g/kg dry matter; DM) and tannins (50–80 g/kg DM) (84).

### Saponins

Saponins are triterpene compounds or steroids found in many crops such as legumes, cereals, tea, and some spices. It is known to have a bitter taste and is toxic at higher concentrations. Saponins affect nutrient absorption by inhibiting some metabolic or digestive enzymes and binding to essential nutrients such as iron, zinc, and vitamin E. Tea saponins have specific characteristics such as strong foaming, emulsifying, dispersing and wetting properties as well as active biological activities, such as anti-cancer, anti-inflammatory, antibacterial, haemolysis, antioxidant, anti-hypertensive, weight loss, nerve stimulation or neuroprotection and fish toxicity, and insecticide activities (1, 85–88). They are found in nature and known to have different biological functions. Saponins, when present with cholesterol, have been reported to show a hypocholesterolemic effect (89). They can cause hypoglycemia, and restrict protein digestion and absorption of vitamins and minerals leading to a leaky gut (90).

### Phytates

Phytates are found in many plants in the form of myo-inositol-1,2,3,4,5,6-hexakis dihydrogen phosphate. They are secondary metabolites that are concentrated naturally in the seeds of legumes, cereals, oil seeds, and all plant-based foods. In the seed, it is stored as phytin or phytate in the husks. Phytates impede the absorption of minerals such as iron, zinc, magnesium, and calcium (90, 91). It can also inhibit the function of digestive enzymes such as pepsin, trypsin, and amylase (92). The negatively charged structure of phytic acid binds to the positively charged ions such as iron, magnesium, calcium, and zinc to make complexes by lower absorption of these ions and thereby reducing their bioavailability. As a chelating agent phytic acid is considered an important anti-nutrient (93).

### Oxalates

Oxalates are oxalic acids present in plants in soluble and insoluble forms. In soluble form, they exist as potassium and sodium salts, and in an insoluble form, it comprises calcium, magnesium, iron salts, or esters. Oxalates are predominantly found in leafy plants and also synthesized in the body. Insoluble forms of oxalates (calcium oxalate) when accumulated in the body lead to harmful effects causing the formation of kidney stones (94). Green leafy vegetables and plants, berries, beans, chocolate, and some nuts contain high amounts of oxalates (95). Although oxalate in normal concentration is easily digestible, people with certain conditions require lower intake as it can lead to complications. In a small population, ingestion of small amounts of oxalate can lead to conditions such as itchy and burning eyes, ears, throat, and mouth, while large amounts may cause stomach upset with nausea and diarrhea (90).

## Effect of anti-nutrients on human health

While the term anti-nutrient is synonymous due to its negative effect, these plant secondary metabolites are known to have many benefits. One has to also consider the effects of concentration as most of the metabolites when consumed in low concentrations do not cause any harm. Anti-nutrients are an integral part of plants as most of them have either a role in plant defense or other functional roles. Anti-nutrients are used as active ingredients in food and drinks, compounds such as saponins, phenolic compounds, and phytic acid when consumed at low levels have shown to lower glucose levels and cholesterol (90). Saponins are also reported to help prevent osteoporosis and maintain liver functions (96). Furthermore, phenolic compounds such as phytic acid, saponins, and protease inhibitors are known to have anti-cancer properties. Meanwhile, tannins are known to have anti-viral, anti-parasitic, anti-bacterial, antioxidant, anticancer, immuno-regulatory, and cardiovascular-protective effects (90, 97). Therefore, it is wise to have knowledge about the constituents of the food products and make a conscious judgment in consuming them. The various beneficial effects of anti-nutrients also help as a valuable tool for managing different diseases. Although not always nutritious, consuming in correct quantities can rather have a beneficial outlook.

## Strategies to mitigate the effects of anti-nutritional factors

Studies have reported the various effects of anti-nutrients on human health by reducing their nutritional significance. Anti-nutritional factors are found widely in plants and plant products, therefore, removing them is essential to enhance their quality. The presence of high amounts of tannins in food products has been shown to affect the bioavailability of iron by inhibiting its absorption and causing deficiency (98, 99). Concurrently, the presence of phytate in foods is known to cause Zinc deficiency (7). Phytate which serves as a phosphate storing molecule in plants has a high affinity to chelate ions such as Zn^2+^, Fe^2+^, Ca^2+^, Mg^2+^, K^2+^, Mn^2+^, and Cu^2+^, thereby reducing the bioavailability and thus affecting growth and development (100). A report on the reduced growth in chicks was reported as a result of the negative impact on the absorption of Vitamin A and E along with lipids in chickens fed with dietary saponins (101).

Anti-nutrients in food are not only known to reduce nutrient utilization causing nutritional deficiency but it is also toxic when consumed at higher concentrations. Therefore, lately, a great interest has been renewed in mitigating this problem. Traditional methods such as soaking, milling, cooking, roasting, debranning, germination, and fermentation have been used since time immemorial for reducing the effects of these anti-nutrient components in foods. Herbal tea is decoctions made out of herbs and is known to contain different types of antinutritional factors in different concentrations. The herbs and medicinal plants used as herbal drinks and studied in the present review contain various phytochemicals including anti-nutrients (Table 2). The antinutritional factors can be reduced during proper processing and correct brewing temperature, boosting the nutritional value by suppressing the antinutritional factors. Here, we describe various processing methods used in reducing the anti-nutrients in these herbal tea products.

## Heating, drying, steaming, and cooking

Heating or dry roasting is used as an application for heat treatments in tea processing. Heating and boiling, among all the methods, are found to be the most effective to reduce harmful compounds in food. Heat treatment of vegetables and cereals leads to the activation of the enzyme phytase making it useful and healthy (102). Drying, an essential thermal process, causes a significant reduction in moisture content, crude protein, crude fiber, ash, and mineral contents, and also the antinutritional factors (flavonoids, alkaloids, glycosides, phenolic compounds, saponins) while it may cause an increase in the oil content. Drying of plants is a step that helps in the prevention of microbial growth while inhibiting biochemical changes, it has been identified to improve the quality of the product due to oxidation reactions (103). Proper drying of *Hibiscus sabdariffa* (roselle) calyxes combined with the right brewing duration of the dried roselle reduces the anti-nutrients and boosts the bioactive content, aroma, and sensory quality (43). Reportedly, lemongrass tea dried in an oven at 40°C was most suitable for the retention of appreciable sensory attributes and least anti-nutrients (63, 64). Drying fresh leaves of *Ocimum tenuiflorum* and processing them as dried, fermented, and unfermented herbal tea showed a differential effect on the phenolic, flavonoid, tannin, and anti-oxidant content, of which the unfermented tea showed an increase in the content in all parameters (103). Although the drying or heating process, an important processing technique, is generally understood to improve the quality of herbal tea, it is yet to understand the chemistry behind the reduction of phytochemical constituents. Therefore, in the herbal tea processing unit drying is an essential step that not only reduces the chances of microbial growth but also enhances aroma and taste due to the release of essential oils. Although heating is considered to reduce the effect of most anti-nutrients, it has been reported that the concentration of polyphenols like tannins increased with an increase in the boiling time (104). Soaking and cooking or boiling is known to greatly impact the nutritional value of food products by reducing their antinutritional contents such as tannins and trypsin (105). Boiling at a controlled temperature for at least 15 min is known to reduce the level of anti-nutrients (106). Similarly, studies on the reduction of phytic acid as a result of soaking and cooking have been reported in legumes (107). A 47% reduction in the content of oxalate in taro leaves was observed as a result of boiling in water for 40 mins, although no significant reduction in oxalate was observed by baking for even 40 min at 180°C (108). Rose petal tea which was oven dried initially at 25–27°C followed by brief drying at 80°C in a hot air oven when brewed in boiling water for 15 min showed good sensory attributes along with higher antioxidative properties (109). Therefore, prolonged boiling should be avoided rather a low time frame for boiling should be followed enough to release sensory properties such as essential oils and aroma which will also help in the reduction of the concentration of polyphenols. As anti-nutrients are not always harmful at lower doses, hence concentration-dependent effects must be considered.

## Fermentation

Fermentation is a process that helps in reducing bacterial contamination in food products. It aids in digestion as it is probiotic and also helps improve the absorption of essential minerals (110, 111). Fermentation of cereal crops in Africa is a common practice as it helps increase nutritional quality (112). It has helped in the increase of essential amino acids such as methionine, tryptophan, and lysine (113). Coulibaly et al. (114) have reported on the significant reduction of phytic acids, tannins, and polyphenols as a result of fermentation in millets. The reduction in phytic acid content could be due to the enzymatic action of the enzyme phytase. The fermentation process also helps in the degradation of phytate due to optimal pH conditions thereby increasing the bioavailability of ions such as iron, zinc, magnesium, and calcium (115). Fermentation is also an important step in the processing of black tea which is known as a source of phytochemicals such as theaflavins and thearubigins that function as potent antioxidants conferring protection against cellular damage. Reports of a popular indigenous beverage of the South African Western Cape prepared from a shrubby legume, Rooibos, which is processed either by air drying or fermented. The herbal tea air dried contains higher levels of polyphenol and thereby contains a rich source of antioxidants which is reportedly higher than green tea (116). The fermented Rooibos tea although did show anti-mutagenic property, however, due to fermentation contains lesser antioxidants, however, the fermented tea showed better sensory attributes as it has a stronger and sweet taste than the unfermented Rooibos tea.

## Genetic engineering

Genetic engineering is a modern technology that has been used to enhance nutritional factors in plants. Using genetic manipulation techniques reduction of phytic acid has been reported in *Zea mays, Oryza sativa, Hordeum vulgare*, and *Glycine max* (117). These genetically modified plants with low phytic acid content could be beneficial for reducing micronutrient malnutrition. Similar reports on the reduction of antinutrients using genetic techniques have been reported in common bean (*Phaseolus vulgaris* L.) seed, which resulted in increased nutrient and iron bioavailability without causing many changes in the agronomic traits (118). Tissue-specific RNAi mediated knockdown of the *ITP5/6K-1* gene in *Oryzae sativa* has reportedly led to a reduction of phytate in seeds (119). Similarly, reports on mutating genes like the *IPK1* gene responsible for producing high levels of phosphorus and stored as phytic acid have been reported in maize (120). While most work on genetic engineering in the reduction of antinutrients has been concentrated on reducing phytic acid which is a major antinutritional factor in legumes and crops (121–123), genome editing techniques are yet to be utilized in knocking out anti-nutrients from other plants. Also, a major impediment to the use of genome editing tools has been the argument of constructing a genetically modified organism which is still not acceptable to many.

It has been observed physical processes (Table 3) like that of heating the plant parts lead to the activation of some essential oils and enhance the aroma. Boiling of herbal tea leads to the reduction of some harmful factors, however, boiling though beneficial has to be followed only for a short duration as a higher time frame leads to concentrated amounts of anti-nutrients due to excessive boiling. Another important process for reducing the harmful effects is found to be fermentation which is known to enhance nutritional quality. As previously described how anti-nutrients affect the bioavailability of micro nutrients and vitamins by lowering their absorption thereby leading to nutritional deficiency, hence it is of utmost importance to reduce the content of these antinutritional factors. Also, apart from reduction strategies, a concerted effort by the consumer to consume the herbal products in moderation as most of these metabolites do not cause major harm if consumed in lower doses.

**Table 3:** Some physical processes for reducing antinutrients.

**Physical processing**	**Comments**
Heating or drying	• Heating at temperatures 40–80°C • Heating or drying prevents microbial growth also reduces chances of formations of aflatoxins
Boiling or brewing	• Mild boiling at low temperatures for around 15 min or brewing leads to higher sensory attributes with higher antioxidative potential
Fermentation	• Increases nutritional quality and reduced polyphenolic content
Genetic engineering	• Gene manipulation techniques can be a good technique to reduce antinutritional factors, however no work in tea or herbal has been reported

## Conclusion

The importance of herbal tea consumption with the growing concern of chronic disorders associated with depleted diets increases. The quality, efficacy, and safety of herbal teas is an area not to be overlooked in future research to develop quality assured processes to harness the goodness of herbs and underutilized plants to be sourced and manufactured to a high standard. The present review attempted a comprehensive study of the underutilized plants as an important source of solutions to the emerging health issues owing to the diet and lifestyle led diseases. Although it is understood that the drying or heating process helps remove anti-nutrients, future research is required on optimizing brewing conditions to understand the synergistic effects of phytochemicals in herbal tea. It is also extremely important for the consumer to consume herbal products in moderation, as most of these metabolites are within a permissible range when consumed in moderation and do not necessarily cause major harm if consumed in lower doses. This review presents the need to explore a wider range of herbal teas, their nutrient, and phytochemical constituents.

## Author contributions

NP and KD conceptualized the idea, designed the outlines, drafted the manuscript, reviewed, and improved the draft. JS, RS, SM, SP, HD, WC, and BW drafted the manuscript. JS, RS, NM, and KD prepared the illustrations and worked on proofreading the manuscript. All authors read and approved the final version of the manuscript.

## Conflict of interest

The authors declare that the research was conducted in the absence of any commercial or financial relationships that could be construed as a potential conflict of interest.

## Publisher's note

All claims expressed in this article are solely those of the authors and do not necessarily represent those of their affiliated organizations, or those of the publisher, the editors and the reviewers. Any product that may be evaluated in this article, or claim that may be made by its manufacturer, is not guaranteed or endorsed by the publisher.
